# Genome-wide identification of novel intergenic enhancer-like elements: implications in the regulation of transcription in *Plasmodium falciparum*

**DOI:** 10.1186/s12864-017-4052-4

**Published:** 2017-08-23

**Authors:** Suyog Ubhe, Mukul Rawat, Srikant Verma, Krishanpal Anamika, Krishanpal Karmodiya

**Affiliations:** 1 Department of Biology, Indian Institute of Science Education and Research, Pashan, Pune, 411008 India; 2 Labs, Persistent Systems Limited, Pingala - Aryabhata, Erandwane, Pune, 411004 India

**Keywords:** Enhancer identification, Histone modifications, Transcription, ChIP-sequencing and gene regulation

## Abstract

**Background:**

The molecular mechanisms of transcriptional regulation are poorly understood in *Plasmodium falciparum*. In addition, most of the genes in *Plasmodium falciparum* are transcriptionally poised and only a handful of cis-regulatory elements are known to operate in transcriptional regulation. Here, we employed an epigenetic signature based approach to identify significance of previously uncharacterised intergenic regions enriched with histone modification marks leading to discovery of enhancer-like elements.

**Results:**

We found that enhancer-like elements are significantly enriched with H3K4me1, generate unique non-coding bi-directional RNAs and majority of them can function as cis-regulators. Furthermore, functional enhancer reporter assay demonstrates that the enhancer-like elements regulate transcription of target genes in *Plasmodium falciparum.* Our study also suggests that the *Plasmodium* genome segregates functionally related genes into discrete housekeeping and pathogenicity/virulence clusters, presumably for robust transcriptional control of virulence/pathogenicity genes.

**Conclusions:**

This report contributes to the understanding of parasite regulatory genomics by identification of enhancer-like elements, defining their epigenetic and transcriptional features and provides a resource of functional cis-regulatory elements that may give insights into the virulence/pathogenicity of *Plasmodium falciparum*.

**Electronic supplementary material:**

The online version of this article (doi:10.1186/s12864-017-4052-4) contains supplementary material, which is available to authorized users.

## Background

Malaria is a life threatening disease caused by a parasite of *Plasmodium* species from apicomplexan genera, with an estimated 200 million cases every year [1]. *Plasmodium falciparum* is the most virulent species with complex life-cycle involving two hosts – humans and mosquitoes. The clinical manifestation of malaria is a result of *Plasmodium’s* growth in red blood cells (RBCs), where it progresses through morphologically and developmentally distinct stages namely ring, trophozoite, and schizont to multiply into 16–32 merozoites to invade fresh RBCs upon bursting. The rapid transition between these morphological states is associated with tight control of gene expression, which is poorly understood in *P. falciparum*.

The haploid genome map of *P. falciparum* laboratory strain 3D7 was published more than a decade ago. Of the 23 Mb nucleotide genome, 53% constitutes coding while the remaining 47% is predicted to be non-coding DNA (ncDNA) [2]. RNA-sequencing data revealed that the gene expression is fine-tuned with the intra-erythrocytic cycle (IEC) [3, 4]. Transcription in eukaryotic organisms is exquisitely orchestrated by concerted interplay of transcriptional regulatory elements hence identification of such regulatory DNA elements has been extensively explored. Numerous regulatory elements such as core promoters, G box, downstream regulatory elements, insulators have been identified while some like TATA box, and cis-acting elements have been identified and proposed to play roles in transcriptional regulation, however, some of them need functional validation [5–9]. Also, substantial amount of research lead to identification of Myb1, HMG box proteins, and ApiAp2 group of transcription factors [10–13].

Transcriptional enhancer elements are one such type of non-coding regulatory DNA sequences that can enhance transcription of target genes regardless of their location and orientation relative to their target promoter by virtue of chromatin looping [14]. Due to the difficulties presented by extremely AT-rich genome and with no fixed physical distance between regulatory elements and the genes they regulate, the task of finding enhancer elements in *Plasmodium* genome has been challenging. To-date only a few enhancer elements are known in *Plasmodium* and their identification has relied on traditional approaches of assaying for differences in functional reporter activities by truncating the DNA sequences [15–17]. Therefore, identification and functional characterisation of enhancer elements from genome by using non-traditional newer approaches is of particular importance to explore the mechanism of transcriptional regulation imposed in *P. falciparum*.

Recently, we generated a comprehensive epigenome map of *P. falciparum* by performing chromatin immunoprecipitation-sequencing (ChIP-seq) for multiple histone modifications at three different IEC-developmental stages – ring, trophozoite, and schizont [18, 19]. Interestingly, we observed significant co-enrichment of multiple histone marks at intergenic regions. The significance of these epigenetic marks at intergenic regions remains elusive. In this study, we identified 462 intergenic enhancer-like elements by analysing their transcriptional-epigenomic status and validated representative enhancers by functional luciferase reporter assays. Thus, our comprehensive analysis of histone modifications identified enhancer-like elements on a genome-wide scale and revealed complex genome architecture that may facilitate spatio-temporal regulation of virulence/pathogenicity genes in *Plasmodium falciparum.*

## Results

### Genome-wide identification of enrichment of histone modifications at intergenic regions

**Fig. 1:**
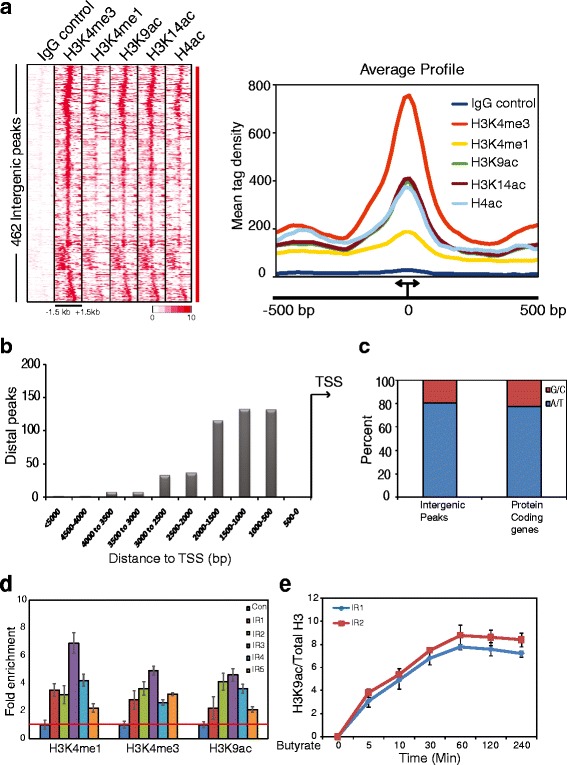
Identification of intergenic peaks which are associated with multiple histone modifications. In order to extract peaks of histone modifications, tag densities of histone modifications were calculated in the IRs as described in Materials and Methods section. **a** Heatmap was generated for the region +/− 1.5 kb around centre of the peak. Tag density of multiple histone modifications over the 462 intergenic peaks (+/− 1.5 kb around centre of the peak) are represented in the heatmap. **b** Distribution of intergenic peaks around the transcription start sites was calculated from the centre of the peaks. The closest peak is 750 bp upstream to TSS as the IRs located within 500 bp upstream of the gene have been excluded from the analysis. **c** AT/GC content of the intergenic peaks and protein coding genes was found comparable as the genome is approximately 80% AT-rich. **d** Presence of H3K4me1, H3K4me3 and H3K9ac over the intergenic regions validated by quantitative ChIP-PCR over randomly selected peaks. The ChIP-qPCR enrichment matches with the tag density per peak (data not shown). **e** Increase in histone H3K9ac over the intergenic peaks following HDAC inhibition by sodium butyrate was measured at indicated time points by ChIP-qPCR. ChIP-signal for histone H3K9ac was normalized to total H3. Control genomic region (primer sequence shown in Additional file 4: Table S1) did not show any increase in acetylation upon sodium butyrate treatment. Error bars represent the standard deviation calculated from three technical replicates

To gain insights into the epigenomic landscape of *P. falciparum*, we used previously generated chromatin immunoprecipitation sequencing (ChIP-seq) data of multiple histone modifications at three different stages of intra-erythrocytic cycle; rings, trophozoites and schizonts [18, 19]. Interestingly, we observed enrichment of histone modifications at the intergenic regions (IRs) apart from promoter regions and gene-body. Role of histone modifications is extensively studied on promoters and in gene body, however, little is known about their role at IRs in the *Plasmodium* genome. Based upon tag density, which defines strength of enrichment of histone modifications (H3K4me3, H3K4me1, H3K9ac, H3K14ac and histone H4ac); we identified 462 IRs (Fig. 1a; Additional file 1: Figure S1 and Additional file 2: Table S4). Interestingly, the profiles of histone modifications have similar pattern (Fig. 1a, left panel) but differential histone modification enrichment (Fig. 1a, right panel). In order to confirm the specificity of the enrichment of histone modifications, we compared publicly available data for H3K4me3 [20] and observed similar pattern (Additional file 1: Figure S2) indicating that both the datasets are of comparable quality and the identified IRs are indeed existent. Furthermore, to confirm identity of the peaks and to rule out the sequence specific biases, we calculated the enrichment of H3K4me3 over pan-H3 on IRs and promoters. Distribution profile and enrichment of H3K4me3 are comparable on these two regulatory elements (Additional file 1: Figure S3), which strengthen our observation that IR peaks are not overrepresented regions of the genome because of sequence biases. As the median intergenic region in *P. falciparum* is <2 kb [21] most of the IRs (80%) are expectedly located 0.75–2 kb upstream to the transcription start sites (TSSs) and remaining within 3 kb distance from the TSSs (Fig. 1b). We also calculated the base-pair (AT/GC) content at IRs and compared it with the promoters of the protein coding genes in order to see if there are any differences. As expected base-pair composition was found comparable at the IRs and protein coding genes (Fig. 1c). Further to validate the distribution of histone modifications at the IRs obtained in ChIP-seq, we performed ChIP-qPCR at the randomly selected loci for H3K4me1, H3K4me3 and H3K9ac. All the selected histone modifications were enriched at IRs, which were validated by ChIP-qPCR (Fig. 1d).

Transcription regulation is coupled by dynamic events of histone modifications mediated by recruitment and enzymatic activities of histone acetyltransferases (HATs) and histone deacetylases (HDACs) at specific loci in genome. About 30–40% of histones can be maintained constitutively in low acetylated states without any functional consequence [22]. So, to investigate if histone acetylation marks at IRs are dynamically regulated, we abrogated the enzymatic activity of PfHDACs type I using a HDAC inhibitor, sodium butyrate [23]. Intra-erythrocytic *Plasmodium* parasites were treated with 10 mM sodium butyrate for indicated time-points and assessed for H3K9ac enrichment relative to total-acetylated H3 over two randomly chosen intergenic regions (IR1 and IR2) by ChIP-qPCR. Within 5 min of HDAC inhibitor (HDACi) treatment, H3K9ac enrichment levels increased by more than 3-fold, and by 60 min a profound increase of about 8-fold was observed (Fig. 1e). This signifies that the histones bound at IRs are certainly dynamically maintained in vivo rather than being constitutively marked by histone modification marks.

### H3K4me1 is significantly enriched on intergenic regions

**Fig. 2:**
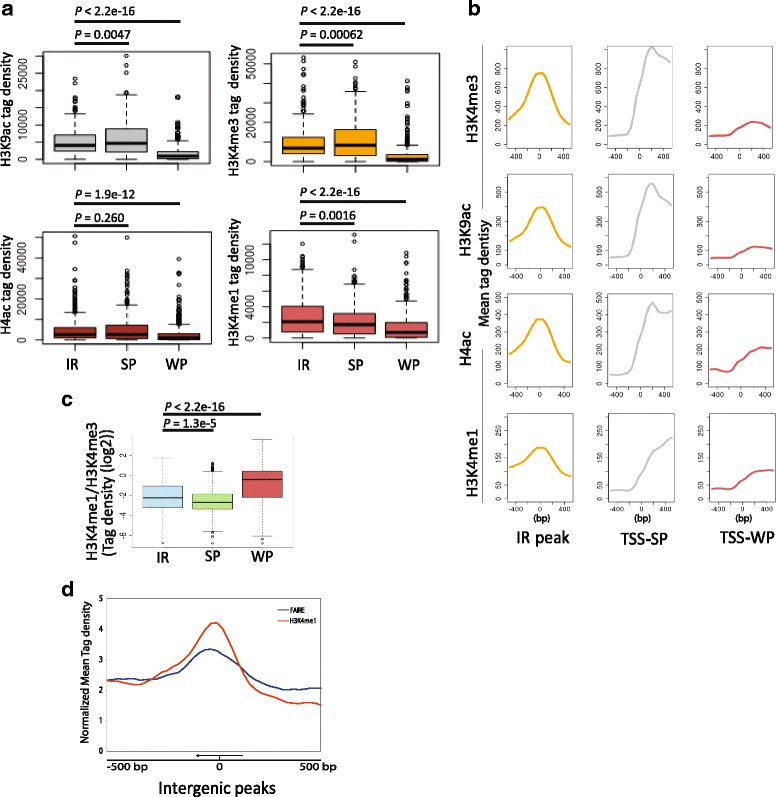
H3K4me1 is significantly enriched at the intergenic peaks. **a** Enrichment of H3K9ac, H3K4me1, H3K4me3 and Histone H4ac over the intergenic peaks and 500 strongly and weakly expressed promoters is calculated. H3K9ac, H3K4me3 and histone H4ac are enriched at the active promoters whereas the distal peaks are enriched with H3K4me1 (*P* < 0.05). IR: Intergenic regions; SP: Strong Promoters; WP: Weak Promoters. *P* values were computed using the Wilcoxon test (two-sided). **b** Profiles of H3K9ac, H3K4me 1, H3K4me3 and histone H4ac over the intergenic peaks and 500 strongly and weakly expressed promoters (+/− 0.5 kb). Intergenic peaks have bell shaped distribution of all histone modifications. **c** One of the characteristic features of the enhancer histone modifications in higher eukaryotes, the ratio of H3K4me1 to H3K4me3 tag density is also higher on intergenic peaks as compared to strongly expressed promoters (*P* < 0.05)**.** Weak promoters showed higher ratio of H3K4me1 to H3K4me3 as compared to IRs, most probably due to absence of active histone modification (i.e. H3K4me3) on weak promoters **d.** Furthermore, one of the hallmarks of the regulatory elements is the nucleosome-depleted regions, which can be assessed by Formaldehyde-Assisted Isolation of Regulatory Elements (FAIRE)-sequencing. To assess if the identified IRs can function in vivo as regulatory elements globally; we integrated available FAIRE-seq data in *Plasmodium* with enrichment of H3K4me1 on identified IRs. Overlapping peaks of H3K4me1 and FAIRE-sequencing over the 462 intergenic peaks suggests that identified IRs are depleted of nucleosomes and that the intergenic peaks are indeed regulatory elements

Histone modification marks are predictive of function of the underlying DNA sequences [24]. We wondered if there is any dominant epigenetic mark at IRs as multiple epigenetic marks co-occur at these intergenic regions. We profiled for H3K9ac, H3K4me1, H3K4me3 and Histone H4ac marks over IRs and compared with 500 strong promoters (promoters of genes with high expression) and 500 weak promoters (promoters of genes with low expression). As expected H3K9ac, H3K4me3 and Histone H4ac, which are known gene activation marks, were enriched at the strong promoters (Fig. 2a and b). Interestingly, IRs were significantly (*p* < 0.05) enriched with H3K4me1, a signature mark of enhancer elements (Fig. 2a and b) in higher eukaryotes [25]. H3K36me3, H3K79me3 and H4K20me3 were found comparable at IRs and strong promoters (Additional file 1: Figure S4). Multiple studies have reported H3K4me3 as a hallmark of active promoters and it was also found to be enriched at active enhancers [26]. Hence, classically ratio of H3K4me1 to H3K4me3 is used to distinguish enhancers from promoter elements [27, 28]. We observed significantly higher ratio of H3K4me1 to H3K4me3 occurring at IRs than that observed for strong promoters, indicating that IRs can putatively function as enhancer elements in *P. falciparum* (Fig. 2c). Surprisingly, weak promoters showed higher ratio of H3K4me1 to H3K4me3 as compared to IRs (Fig. 2c), most probably due to absence of active histone modification (i.e. H3K4me3) over weak promoters (Fig. 2a). Intergenic regulatory elements are found to be enriched for acetylation marks at H3K9 and H3K14 (Figs. 1a and 2a) as reported earlier [29, 30]. Another hallmark of the regulatory elements is the nucleosome-depleted regions, which can be assessed by Formaldehyde-Assisted Isolation of Regulatory Elements (FAIRE)-sequencing. To assess if the identified IRs, which are also marked by H3K9ac and H3K14ac, can function in vivo as regulatory elements globally; we integrated available FAIRE-seq data in *Plasmodium* [31] with identified IRs. Interestingly, the identified IRs are depleted of nucleosomes indicating that the predicted IRs are indeed regulatory elements (Fig. 2d).

### H3K4me1 enriched intergenic regulatory elements exhibit orientation-independent enhancer reporter activity

**Fig. 3:**
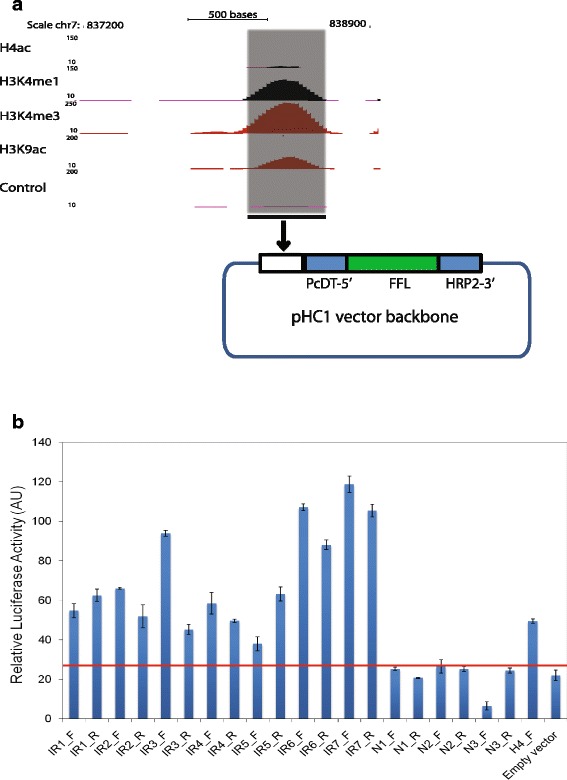
H3K4me1 marked intergenic peaks exhibit enhancer activity in enhancer luciferase assay. **a** UCSC snapshot of a representative intergenic peak having multiple histone modifications. The intergenic region (~ 1 kb) was cloned in an enhancer luciferase reporter vector. **b** Enhancer reporter (luciferase) assay was carried out to measure the activity of seven randomly selected intergenic regions (IR1 to IR 7) and compared to randomly selected negative genomic regions (N1-N3) and empty vector control in *P. falciparum*. F denotes forward orientation, whereas its opposite orientation (Reverse) is denoted as R. Earlier reported enhancer (H4) [15] was used as positive control. Intergenic regions showed enhancer activity in the enhancer luciferase assay independent of orientation. The red line indicates the activity obtained with control negative genomic region. Error bars represent the standard deviation for two biological replicates

To verify the enhancer function of the intergenic regulatory elements, we developed an enhancer luciferase vector by using pHC1 vector as a backbone [32]. Firefly luciferase (FFL) gene was cloned in pHC1 between 5′ PcDT promoter and 3′-HRP2 termination signal (Fig. 3a). Randomly selected IR sequences 1 to 7 with putative enhancer properties, negative controls N1 to N3 (randomly selected genomic regions without any histone modifications) were amplified from *P. falciparum* genomic DNA and cloned in pHC1-enhancer luciferase vector upstream of luciferase gene and PcDT 5′ promoter (Fig. 3a). DNA sequence confirmed clones were used for transient transfection assays in *P. falciparum* by supplementing parasites with DNA-loaded RBCs [33]. Parasites were cultured for 2 passages and then assayed for luciferase activity as per manufacturer’s instructions. The tested IRs (IR 1 to 7) showed enhancer luciferase activity comparable to previously reported enhancer element (H4) of the gene histidine-rich protein 3 (hrp3) from *P. falciparum* (Fig. 3b) [15]*.* Moreover, luciferase activity was also detected in reverse orientation, whereas luciferase activity in negative controls (N1 to N3), regions lacking enhancer-like properties, was comparable to empty vector control. Exhibition of enhancer luciferase activity by IRs in either orientation suggests that enhancer activity is orientation independent. Thus, the functional enhancer reporter assay demonstrated that IRs may function as enhancer-like elements to regulate transcription of target genes in *Plasmodium.*

### Enhancer-like elements generate non-coding transcripts which positively correlate with the transcription of nearest gene

**Fig. 4:**
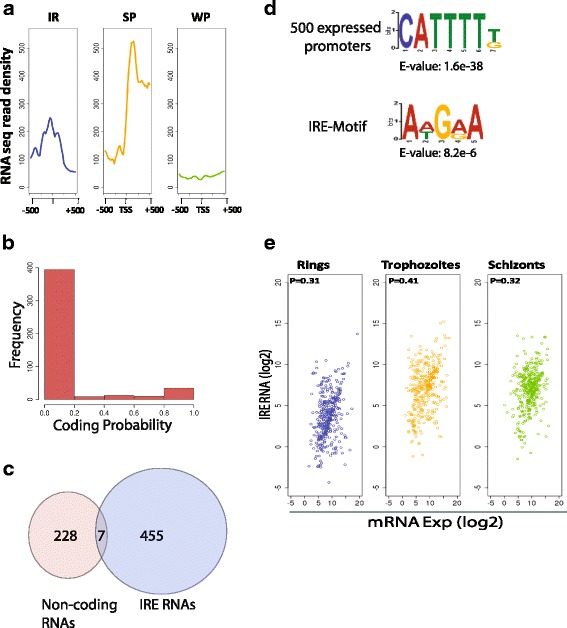
Enhancer-like intergenic peaks produce short RNAs which positively correlates with the expression of nearest gene. **a** Distribution of RNA-sequencing reads is plotted over the intergenic peaks and 500 strongly and weakly expressed promoters. Average distribution of RNA-sequencing reads suggests that intergenic peaks produce short (150–300 bps) bi-directional transcripts. **b** Most of the enhancer-like element RNAs do not exhibit protein coding potential. **c** Only 7 of the enhancer-like element RNAs overlap with known non-coding RNAs suggesting that enhancer-like element RNAs are novel non-coding RNAs. **d** The consensus motif at the enhancer-like element is different than the motif found at the highly expressed promoters indicating involvement of different set of transcription factors. **e** Correlation in the expression of enhancer-like element RNAs from the intergenic peaks is calculated with the expression of nearest gene in a stage specific manner. Enhancer-like element RNAs produced from intergenic regulatory peaks showed moderately positive correlation with the expression of nearest neighbourhood gene

Transcription is a characteristic feature of enhancer elements by virtue of RNA Polymerase II occupancy [34–36]. *Plasmodium* enhancer-like elements are also co-enriched with H3K4me3, H3K9ac and H4ac (Fig. 2a and b) and H3K36me3, H3K79me3 (Additional file 1: Figure S4) epigenetic marks, which are indicative of active transcription at these elements [18]. So, we mapped RNA-sequencing reads to *Plasmodium* genome to interrogate the transcriptional output from 462 enhancer-like elements. For comparison, we also included RNA-seq reads from 500 strong and weak promoters each, respectively in our analysis. Consistent with observation in higher eukaryotes, transcription of 150–300 bps was observed from enhancer-like elements, while transcription proceeded uni-directionally towards termination site from strong promoters and no transcription was detected from weak promoters (Fig. 4a). Production of non-coding RNAs from IRs is also verified by qRT-PCR over randomly selected loci (Additional file 1: Figure S5).

Recently, a comprehensive map of transcription start sites (TSSs) was reported in *Plasmodium falciparum* [37]. We observed an overlap of 8 genomic regions between the current set of 462 strong peaks (considering a region of 250 bp up/down of a peak centre) and 1580 newly reported TSSs (Additional file 3: Table S5). Six of these overlapping enhancer-like elements are smaller than 115 nucleotides indicating that ORFs produced from them are very small (<30 amino acids), which in turn suggest that they might be acting as enhancer-like elements rather than promoter. Nevertheless, non-overlap between 454 intergenic peaks reiterates that the regions we have identified are not unannotated promoters. We wondered if the RNA produced from enhancer-like elements has protein coding potential and the identified intergenic regions are in-fact unannotated promoters. We first tested the accuracy of the known tools for predicting coding potential of a given nucleotide sequence from *P. falciparum*. A total of 5536 non-redundant protein coding and 1563 non-coding RNA sequences (snRNA, tRNA, rRNA, snoRNA and miscellaneous RNA from all *Plasmodium* species) were extracted from PlasmoDB (http://plasmodb.org). Coding Potential Calculator (CPC) was found to predict 100% known non-coding RNAs from *Plasmodium* (Additional file 1: Figure S6). Coding potential of enhancer-like elements by CPC suggest that more than 90% of the identified intergenic elements have no protein coding potential (Fig. 4b). Interestingly, only 7 of the 462 enhancer-like element RNAs overlap with known non-coding RNAs (Fig. 4c). Furthermore, the most frequently occurring motif enriched at enhancer-like element sequences is significantly different than the motifs enriched at the promoters of highly expressing genes (Fig. 4d) and randomly selected intergenic regions (Additional file 1: Figure S7). TomTom search identified that the enhancer-like element motif – AWGRA has similar recognition sequence as found for some of the ApiAp2 TFs namely PF11_0404_D1, PFL1900w_D1, and PF13_0267; but as indicated the E-values are higher reducing the confidence-level (Additional file 1: Figure S8). Collectively, these indicate that the enhancer-like element RNAs are novel non-coding RNAs.

In higher eukaryotic systems enhancers are shown to regulate the transcription of nearest neighbourhood gene and their RNA (eRNA) level correlates with expression of the nearest gene [35]. We wondered if it is also true for enhancer-like element RNAs and so we calculated the correlation in expression between enhancer-like element RNAs and nearest neighbourhood gene expression. The expression of transcribed enhancer-like element RNAs correlates positively with the expression of nearest neighbouring genes at ring, trophozoite and schizont stages of IEC as evident from positive Pearson correlation coefficient values 0.31, 0.41, and 0.32, respectively (Fig. 4e). The moderately positive Pearson correlation coefficients between different stages indicate presence of additional layers of contacts playing a role in stage specific transcriptional regulation. This in turn suggests that enhancer-like element RNAs could have a functional role in regulating transcription.

### Enhancer-like elements are clustered spatially over virulence-pathogenicity genes

**Fig. 5:**
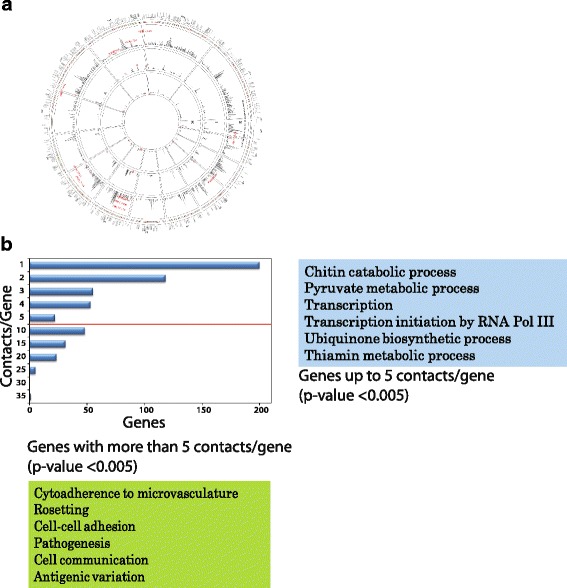
Enhancer-like intergenic peaks form multiple contacts in the genome and exhibit stage specific functions. **a** Circular plot represents number of contacts made by the *P. falciparum* genes with enhancer-like elements and their correlation with gene expression. Overall, enhancer-gene body contacts increase from rings (R) to trophozoites (T) to schizonts (S) stage. The inner three tracks labelled with S, T and R show bar plots for the number of contacts per gene in the particular stage. There are total 959 genes (includes 11 virulence genes) contacting enhancers with maximum number of contact made by a gene is 58. Out of 959 genes, 366 genes have strong positive or negative correlation between the number of contacts and gene-expression. These are shown as colored dots along with the gene label on each chromosome from pf1 to pf14 and MT (mitochondrial) in the outermost two tracks. The color codes for the dots are: Dark green represents correlation value ranging from −1.0 to −0.9; Green represents correlation value ranging from > −0.9 to −0.7; Orange represents correlation value ranging from 0.7 to <0.9; Red represents correlation value ranging from >0.9 to 1.0. Interestingly, the virulence genes (represented by red circles in each track S, T and R) show highest enhancer contacts in T stage. These virulence genes are labelled in red texts. 6 out of 11 virulence genes (PFB0010w, MAL7P1.50, PF08_0141, PF08_0103, PFL0935c and PF13_0003) show strong positive correlation (> = 0.7) between the contacts made by them and their expression. The correlation between number of contacts of genes with enhancers and its expression across three stages is calculated using Pearson method. **b** Number of contacts established per gene by enhancer-like elements is calculated for trophozoite stage. Gene ontology of genes showing more than 5 contacts and equal to or less than 5 contacts per gene. Genes with more than 5 contacts are mostly involved in the complex regulation pathways however; genes showing equal to or less than 5 contacts are involved in the maintenance of housekeeping function

Eukaryotic genomes are organised spatially into higher order structure to accommodate chromatin in smaller nuclear space. The three-dimensional genome architecture is defined by its physical division into topological domains within which chromatin looping mechanisms mediate enhancer-promoter interactions [38]. In order to investigate the topological contacts of enhancer-like elements globally, we mapped Hi-C data generated at 10 kb resolution of the spatial organization of *P. falciparum* genome at three different stages [39]. Enhancer-like elements are involved in intra-chromosomal (contacts formed within same chromosome i.e. cis-interactions) as well as inter-chromosomal (contacts formed with regions from another chromosome i.e. trans-interactions) interactions with gene promoters. Cis-interactions of enhancer-like elements with gene promoters are more ubiquitous than trans-interactions (Fig. 5a). We filtered the Hi-C data to get contact information of the 462 enhancer-like elements with the genes falling within 1 kb of the target promoter regions. The total number of such genes is 959. Overall, number of contacts increased from rings (1203) to trophozoites (2247) to schizonts (9732) indicating either tighter mechanical packaging of chromatin or stage-specific dynamic role of enhancer-gene contacts. Furthermore, we have taken equal number (462) of random non-enhancer like genomic sequences and performed the analysis for contact information. As the resolution of the Hi-C data is 10 kb and chromatin packaging varies across the different stages, we observed linear increase in contacts from the rings to trophozoites to schizonts indicating tighter mechanical packaging of chromatin during parasite growth. However, random non-enhancer like sequences do not show higher contacts per gene suggesting that higher number of contacts formed by enhancer-like sequences are specific (Additional file 1: Figure S9). Moreover, on an average, an active enhancer-like element contacts 9.5 promoters while a promoter contacts 4.5 enhancers, suggesting enhancer-like elements are clustered spatially with multiple genes in nucleus (Fig. 5a). More number of contacts has been established for the virulence genes than other genes in *Plasmodium* genome [39, 40], additionally, our study showed that genes with more than 5 contacts are involved in *Plasmodium* pathogenesis like cytoadherence and rosetting while genes with 5 or less contacts are involved in housekeeping functions (Fig. 5b). The biological implications for segregation of genes into housekeeping and virulence-pathogenicity clusters point towards the existence of differential transcriptional control. Clustering of virulence genes has been shown by DNA fluorescence in situ hybridization (FISH) in previous studies [41, 42]. Here, we hypothesize that clusters with more number of interactions among enhancer-like elements and genes fine-tune the transcriptional program for co-regulated control over virulence-pathogenicity gene expression. Though further experimentations are required, the observations highlight involvement of genomic context i.e. nuclear architecture, micro-environment within transcriptional clusters like availability of some nuclear factors (e.g. transcription factors, chromatin remodellers) in enhancer functionality.

## Discussion

Identification of enhancer elements in *P. falciparum* has been challenging largely due to their property to work in location-, orientation- independent manner; additionally AT-richness of *Plasmodium* genome makes it difficult to identify regulatory elements [43]. Furthermore, functional validation of identified regulatory element is difficult as manipulation of *P. falciparum* genome is hard to achieve. In this study, we have integrated ChIP-seq data sets for multiple histone modification marks to identify and characterize novel enhancer-like elements with their potential role in transcription regulation in *P. falciparum*.

### Intergenic sequences are enhancer-like elements

We illustrate that the identified intergenic regions exhibit features similar to those observed at transcriptional enhancer elements in higher eukaryotic systems. The assurance about identified enhancer-like elements not being unannotated promoters is supported by multiple observations as stated herein. First, a distinctive epigenetic profile, H3K4me1 as the significantly enriched histone modification mark and higher H3K4me1/H3K4me3 ratio persists at enhancer-like elements in comparison to promoters [27, 28]. Second, the intergenic regions displayed orientation-independent enhancer activity in transient firefly luciferase assay confirming their functionality as enhancer elements in *P. falciparum*. Third, characteristic bi-directional transcription is observed from enhancer-like elements [44, 45] while transcription proceeds uni-directionally from promoter elements (Fig. 4a). Furthermore, the transcripts generated from enhancer-like elements are non-coding RNAs (Fig. 4b and c). And fourth, distinct motif is enriched in enhancer-like elements than at promoters (Fig. 4d). In higher eukaryotic systems, classical histone marks of enhancers are H3K4me1, H3K4me3, H3K27ac and H3K27me3 [46, 47]. Acetylation and methylation of H3K27 permit to distinguish between active and inactive/poised enhancers, respectively. Interestingly, *P. falciparum* lacks H3K27me3 [18, 48] and seems to have very low levels of H3K27 acetylation [18], suggesting that identified intergenic regions are enhancer-like elements with a potential distinct signature in *P. falciparum*.

### Intergenic regulatory element RNAs- a novel class of non-coding RNAs which positively correlates with expression of nearest neighbourhood genes

The chromatin state at enhancer-like elements appears to be transcriptionally active owing to nucleosome-depleted region [18, 31]. Comprehensive epigenomic mapping of regulatory elements revealed that multiple histone modification marks persist at enhancers in higher eukaryotic systems [49–51]. Similarly, the genomic loci of enhancer-like elements are enriched with multiple activations marks (eg. H3K4me3, H3K9ac etc) in *P. falciparum* [18, 41]. Thus, enhancer-like elements also gain transcriptionally active states equivalent to that of coding regions and produces RNAs. These enhancer-like element RNAs, which are equivalent to enhancer RNAs (eRNAs) in higher eukaryotic system, are a new class of non-coding RNA in *Plasmodium* that are transcribed bi-directionally from enhancer-like elements. Interestingly, bi-directionally transcribing enhancer RNAs have also been used to predict active enhancers [34, 52]. Experiments from mammalian cell lines implicate eRNAs in a variety of functions such as chromatin looping, recruiting histone acetyltransferases, augmenting chromatin accessibility, depositing H3K4me1/2 marks at enhancers, and increasing recruitment of RNA Polymerase II at gene promoters [43, 44]. As of now, we have no interpretation for the biological functionality of enhancer-like element RNAs, but their expression positively correlates with the expression of the nearest neighbouring gene along IEC of *P. falciparum* implying that a substantial proportion of enhancer-like elements can harbour their target genes in immediate vicinity and can act by mechanisms involving enhancer-like element RNAs. Deletion of the enhancer-like elements in the genomic context should provide final evidence of functional activity. Unfortunately, generating transgenic lines in *P. falciparum* is non-trivial, limiting the use of modern techniques in deciphering the role of enhancer-like elements. Since, most of the genes are poised in *P. falciparum* [18], further systematic investigation of the function of enhancer-like element RNAs should reveal the diverse transcriptional regulatory mechanisms that orchestrate *P. falciparum’s* life cycle and may identify novel mechanisms that can be targeted for future drug-development against malaria parasite.

### Intergenic regulatory elements, spatial genome organization and regulation of clonally variant multicopy genes

Epigenetic profile at enhancer-like elements does not change drastically along the developmental stages of parasite suggesting an additional layer of transcriptional regulation in *P. falciparum*, which is different than stimulus-dependent enhancer activation in higher eukaryotic systems [53, 54]. We postulate that the dynamic global genome re-organisation events during IEC participate in spatially organising the enhancer-like elements that lead to establishment of their interactions with target gene promoters [31, 39]. In genome-wide nuclear architecture context, enhancer-like elements are involved predominantly in intra-chromosomal than inter-chromosomal interactions with genes. This observation is consistent with earlier report that majority of DNA interactions are contained within intra-chromosomal structures [39, 55]. Enhancer-like elements are involved in spatial clustering with multiple genes in three-dimensional nuclear architecture. *Plasmodium* genome is known to be partitioned into active and repressed zones for transcriptional regulation of virulence genes [56] and active transcription occurs at few discrete locations that appear to be developmentally regulated along the IEC of *Plasmodium* [42]. Numerous studies suggest that genomic loci of virulence-pathogenicity genes, which are clonally variant multicopy (CVM) genes, possess characteristically distinct epigenetic features. CVM genes are particularly marked by histone H4 acetylation activation mark and H3K9me3 repression mark [18, 41]. Involvement of this combinatorial pattern of epigenetic marks in segregating genes into housekeeping and virulence-pathogenicity clusters needs further investigation. Identification of molecular players involved in segregating and maintaining functionally linked genes as clusters would enable genetic manipulations to disrupt clusters of enhancer-like elements and virulence-pathogenicity genes. Genes with more than 5 contacts are all involved in mechanisms of pathogenesis like rosetting, cytoadherence and virulence; while those with 5 or less contacts are associated with genes of housekeeping functions. The biological implications for segregation of genes into housekeeping and virulence-pathogenicity clusters point towards the existence of differential transcriptional control. Clustering of virulence genes (2 to 5 clusters per cell) have been shown by DNA fluorescence in situ hybridization (FISH) in previous studies [39, 55]. Here, we hypothesize that clusters with more number of interactions among enhancer-like elements and genes fine-tune the transcriptional program for co-regulated control over virulence-pathogenicity gene expression. Though further experimentations are required, the observations highlight involvement of genomic context i.e. nuclear architecture, micro-environment within transcriptional clusters like availability of some nuclear factors (e.g. transcription factors, chromatin remodellers) in enhancer functionality.

*Plasmodium* codes for Apicomplexan Apetala2 (ApiAp2) family of proteins and are the major known transcription factors in genome [10]. Genome-wide DNA-binding motifs have been validated for ApiAp2 transcription factors and members of ApiAp2 family are known to bind numerous distinct motifs apart from the highly preferred primary motif. The complexity increases further as ApiAp2 TFs can form homo- and hetero-dimers [57–60]. The enhancer-like element motif - AWGRA seems to be a sequence extension of ApiAp2 core motifs namely PF11_0404_D1, PFL1900w_D1, and PF13_0267 plausibly suggesting their involvement in transcriptional control at enhancer-like elements. The transcriptional co-activator PfGCN5 along with its acetylated target H3K9/K14 has been reported to be enriched at a previously known pf1-cys-prx enhancer element [16, 61]. Our data demonstrates that the nucleosomes positioned at identified enhancer-like elements are marked by H3K9 and H3K14 acetylation, implicating PfGCN5 as a transcriptional co-activator at enhancer-like elements. Assembly of protein complex at enhancers called the enhanceosome complex requires acetylation by GCN5, which eventually leads to recruitment of transcription machinery [62]. Future research on *Plasmodium* enhanceosome will aid in our understanding of the concerted action of sequence- and/or structure-specific transcription factors, chromatin modifiers and remodelers that participate in transcriptional regulation mediated by enhancer-like elements.

## Conclusions

In conclusion, this study identified genome-wide intergenic regulatory elements in *P. falciparum* that function as transcriptional enhancers. As observed in higher eukaryotes such as human and mouse, intergenic regulatory elements produce non-coding RNAs, which positively correlate with the gene expression. Even though the function of these elements remains to be established individually in *P. falciparum*, our study is the first report in any lower eukaryotic parasitic system where the enhancers are characterized in such greater details. Thus, this report contributes to the fundamental understanding of parasite regulatory genomics by first time identification of novel genome-wide enhancer-like elements, defining their epigenetic and transcriptional features and provides a resource of functional cis-regulatory elements that may give insights into the virulence/pathogenicity of *Plasmodium falciparum*.

## Methods

### Culturing of *Plasmodium falciparum* parasites

*P. falciparum* culture-adapted parasite lines were kindly provided by Dr. Vasudevan Seshadri (National Center for Cell Science, Pune) and were cultured as previously described [63]. Briefly, parasites were cultured at 10% parasitemia in RPMI1640 medium (Pan-Biotech, Germany) supplemented with 25 mM HEPES, 0.5% AlbuMAX I, 1.77 mM sodium bicarbonate, 100 μM hypoxanthine and 12.5 μg ml^−1^ gentamicin sulphate at 37 °C. Parasites were subcultured by replacing the entire spent medium and splitting the culture into two or more flasks to maintain parasitemia to 10% and quickly restoring the hematocrit to 1% in the required volume of culture medium.

### Data source and analysis

ChIP-seq datasets were downloaded from the public data bank Gene Expression Omnibus (http://www.ncbi.nlm.nih.gov/gds) under the accession number GSE63369 (H3K4me3, H3K4me1, H3K9ac, H3K14ac, H4ac, Pan-H3). ChIP-sequencing data for histone modifications (H3K36me2, H3K36me3 and H4K20me3) and RNA-sequencing (GSE23865) were downloaded from the public databank Gene Expression Omnibus for *P. falciparum* [64, 65]. Chromosome conformation capture coupled with next-generation sequencing technology (Hi-C) contact data were used from earlier study [39]. GSE50199 (GSM1215592, GSM1215593 and GSM1215594) for co-ordinates for contacts from Hi-C at ring, trophozoite and schizont stages, respectively. Formaldehyde-assisted isolation of regulatory elements to extract protein-free DNA (FAIRE) data (SRR030738) was integrated from earlier study [31]. The scatter plots, k-means clustering and average gene profiles were created using seqMINER [31, 66]. Box plots and correlation analysis were produced using ‘R’ software (http://r-project.org/). Sequences of primers used for quantitative ChIP-PCR validation are listed in Additional file 4: Table S1.

### Establishment of list of intergenic regions enriched for histone modifications

Transcription start sites (TSSs) and transcription termination sites (TTSs) of coding gene in *P. falciparum* were determined as described earlier [18]. Intergenic regions were selected by excluding gene unit (from TSS to TTS as described earlier [18] and +/− 500 bp around gene unit. A total of 462 histone modification peaks were identified in the entire *P. falciparum* intergenic genome using seqMINER [66].

### Visualization of histone modifications on intergenic regions

We extracted the tag density in a 1.5 kb window surrounding the intergenic peaks using the program seqMINER which generates heatmap as well as the profiles [66]. The sequenced ChIP-seq reads represent only the end of each immunoprecipitated fragments instead of the precise protein-DNA binding sites. To illustrate the entire DNA fragment, essentially before analysis, 3′ end of each ChIP-seq read was extended to 200 bp in the direction of the reads. Data is normalized by dividing the reads per bin by total reads per modification.

### Distance plot calculation

Distance from Transcription Start Sites (TSSs) for each intergenic peak was calculated from the centre of the intergenic peak to the nearest TSS. The distribution of peaks is plotted for every 500 bps around the TSSs.

### Calculation of average length of intergenic peaks

P. falciparum3D7_Genome_v9.3 was used to calculate the length of all the genes in *P. falciparum*. Length of intergenic peaks is calculated by taking the mean tag density over the intergenic peaks in a window of +/− 1000 bps. Background tag density was used to put an arbitrary cut-off for determining the average intergenic peak length.

### Calculation of distribution of histone modifications marks over intergenic peaks and promoters

Total tag density of histone modifications was calculated as described above in average profile calculation over the strong intergenic peaks, 500 strongly and weakly expressed promoters. The profiles of histone modifications over the intergenic peaks, strongly and weakly expressed promoters are calculated using seqMINER [66].

### Construction of luciferase vector system to test the enhancer activity of intergenic peaks

pHC1 vector (obtained from BEI resources) was used as a template to develop a enhancer luciferase system for *Plasmodium falciparum*. pHC1 vector was first digested with the HindIII restriction enzyme to remove the extra set of promoter and 3′ UTR (CAM5’ promoter and HSP86–3′ UTR) and re-ligated using T4 DNA ligase. Later Tg-DHFR-TS gene was replaced with the Luciferase gene from pGL3 promoter vector (Promega) and resultant vector is called pHC1 enhancer vector. Predicted intergenic sites enriched with H3K4me1 and negative regions devoid of any regulatory marks (~1 kb) were amplified from *Plasmodium* genomic DNA and cloned into pHC1 enhancer vector in single HindIII restriction site upstream of luciferase gene and PcDT 5′ promoter. Confirmation of orientation was performed by restriction mapping and DNA sequencing. Co-ordinates of the cloned sites and primer information are provided in Additional file 1: Table S2.

### Enhancer luciferase reporter assay

Equal amount of parasites were cultured on RBCs loaded with indicated DNA constructs for 2 passages. Culture was spun at 1500 rpm for 5 min, and the pellet subjected to saponin lysis in 1X PBS for 10 min at 4^0^ C. Parasite pellets obtained by centrifugation at 2800 rpm for 5 min were washed with 1X PBS to remove haemoglobin. Parasites were lysed in 30 uL of 1X Passive Lysis Buffer by incubating on ice for 10 min. Luciferase assay was performed with Promega Kit as per instructions. Lysates were spun at top speed for 3 min and 20 uL of supernatant is added in 100 uL of Luciferase Assay Reagent II (LAR II) and firefly luciferase activity measured luminometrically. Luminescence Units were expressed as fold change above the empty vector control and also normalised with parasitemia counts measured at the time of harvesting. Experiments were performed at least twice.

### Processing of RNA-seq reads to visualize the distribution over the intergenic peaks and promoters and its correlation with expression of nearest neighbourhood gene

Genes were categorized based on increasing expression levels (based on reads assigned per kilobase of target per million mapped reads (RPKM)) using the RNA-seq data (GSE23865). Datasets for different stages were normalized for the total number of uniquely mapped tags. Stage specific RNA-Seq data was corrected for each stage by a correction factor (based on the amount of RNA per parasite nucleus) present in each stage as described earlier. Distribution of RNA-seq reads over the intergenic peaks, and 500 strongly and weakly expressed promoter is calculated using the seqMINER [66]. Correlation between the RNA produced from the intergenic peaks is calculated with the expression of nearest neighbouring gene (closest distance from transcription start site irrespective of upstream or downstream of the gene unit).

### Circular plot

Circular plot was generated in R v3.2.2 using circlize v0.3.5 package of CRAN. The genomic interactions observed in Hi-C data [39] were filtered at FDR < 0.01. The filtered dataset has 7830 interactions in ring stage (Additional file 5: Table S3 Rings_contact_FDR0.01), 18,320 interactions in trophozoite stage (Additional file 5: Table S3 Trophozoites_contact_FDR0.01) and 65,534 interactions in schizont stage (Additional file 5: Table S3 Schizonts_contact_FDR0.01). The contact information is extracted as described: let us assume a scenario where a genomic locus GL1 interacts with another genomic locus GL2 (both from Hi-C data), a Peak P1 (from ChIP-seq data) overlaps with GL1, and a Gene Y is within 1 kb to GL2. Thus, it indicates that Peak P1 is interacting with the Gene Y. Likewise, we determined all the overlapping genomic loci GL1 for each peak P1 considering there is at least 50 bp overlap between 250 bp up- and down-stream of the peak centre and 5 kb up- and down-stream of the genomic locus. We considered only those Peak (P1)-Gene(Y) interactions where distance between Gene Y and GL2 is <1 kb. There are 1203 such interactions in Ring stage, 2247 interactions in Trophozoite stage and 9732 interactions in Schizont stage (Additional file 5: Table S3 Rings_1kb_contacts, Trophozoites_1kb_contacts and Schizonts_1kb_contacts). For each gene Y, all the enhancer contacts (P1-Y interactions) are counted (Additional file 5: Table S3 Rings_Enhancer_contacts_perGene, Trophozoites_Enhancer_contacts_per Gene and Schizonts_Enhancer_contacts_per Gene). We have used RNA-Seq data (GSE23865) to get the gene-expression values for the genes (Additional file 5: Table S3 Exp_RTS). The correlation between enhancer contacts and gene-expression values for each gene is calculated in R using Pearson method (Additional file 5: Table S3 Count_Exp_cor). For this analysis, we have used PlasmoDB-9.3_P. falciparum 3D7_Genome (http://plasmodb.org/common/downloads/release-9.3/Pfalciparum3D7/).

### Calculation of AT/GC content

Nucleotide sequence of the 462 enhancer-like elements were prepared by fetching 250 bases up and down of the peak centre using P. falciparum3D7_Genome_v9.3, and per base GC content and per sequence GC content was calculated. The average GC% in 5398 protein coding genes (downloaded from www.plasmodb.org) and the 462 enhancer sequences were 22.867 and 20.949, respectively. GC% of a sequence was calculated by dividing the number of G or C nucleotides in the sequence by the sequence length, and multiplying with 100. Similarly, the GC% at a base position in the enhancer sequences was calculated by dividing the number of G or C nucleotides at the position by the number of enhancer sequences accounted for that position, and multiplying with 100.

### Motif analysis

Consensus sequence was determined using MEME-ChIP (meme-suite.org/tools/meme-chip). It is a comprehensive motif analysis tool for large nucleotide sequences. Input DNA was MALARIA (*Plasmodium falciparum*) DNA and database was Malaria (Campbell et al. [59]). The program was run using default parameters.

### Prediction of coding potential of the sequences

Tools tested for predicting coding potential of given sequence are CPC (Coding Potential Calculator; http://cpc.cbi.pku.edu.cn/), PLEK (Predictor of Long non-coding RNAs and messenger RNAs based on an improved k-mer scheme; http://sourceforge.net/projects/plek/), CNCI (Coding-Non-Coding Index; https://github.com/www-bioinfo-org/CNCI) and CPAT (Coding Potential Assessment Tool; http://lilab.research.bcm.edu/cpat/index.php).

### Chromatin immunoprecipation (ChIP) and ChIP-qPCR

Chromatin immunoprecipitation (ChIP) and ChIP-qPCR are performed as described previously [18, 67]. Infected RBCs were cross-linked with 1% formaldehyde (Catalogue number- 28908, THERMO Scientific) for 10 min, lysed and sonicated. ChIP samples were reverse-crosslinked and DNA was purified using a Qiaquick column (Qiagen). Target sites obtained from ChIP-seq analysis were further validated by quantitative PCR using Power SYBR Green Master Mix (Applied Biosystems).

### Sodium butyrate treatment of *P. falciparum*

Parasites were treated with 10 mM sodium butyrate for 0, 5, 10, 30 min, 1, 2, 4, 8 and 16 h. Parasites were cross-linked and ChIP was carried out as described above using H3K9ac antibody [18]. Levels of acetylation were compared by quantitative real-time quantitative PCR using Power SYBR Green Master Mix (Applied Biosystems).

### Gene ontology analysis

Gene ontology analysis was performed using MADIBA [68]; an online web interface (http://madiba.bi.up.ac.za/).

## Additional files

Additional file 1: Supplementary Figures (S1-S9). (DOCX 2070 kb)

Additional file 2: Table S4. List of the enhancer-like elements identified in *Plasmodium falciparum*. (XLS 71 kb)

Additional file 3: Table S5. Overlap between New TSS List from Adjalleyet. al., 2016and the enhancer-like elements identified in this study. (XLS 19 kb)

Additional file 4: Table S1. Primers used for ChIP-qPCR are provided as a separate excel-file. **Table S2.** Co-ordinates and primers used for the cloning of Intergenic regions for luciferase reporter assay are provided as a separate excel-file. (XLSX 12 kb)

Additional file 5: Table S3. Contact information for ring, trophozoite and schizont stages is provided as a separate excel-file. (XLS 11820 kb)

## Abbreviations

ChIP Seq: Chromatin immunoprecipitation sequencing
eRNAs: enhancer RNAs
FAIRE Seq: Formaldehyde-assisted isolation of regulatory elements sequencing
HATs: Histone acetyl transferases
HDACs: Histone deacetylases
IEC: Intra-erythrocytic cycle
IRs: Intergenic regions
ncDNA: Non coding DNA
ORFs: Open reading frames
RBCs: Red blood cells
RPKM: Reads per kilobase per million
TSS: Transcription start site
TTS: Transcription termination site
