# Supplementary material for: Genome-wide identification of novel intergenic enhancer-like elements: implications in the regulation of transcription in Plasmodium falciparum
Source: BMC Genomics. 2017 Aug 23;18:656. doi: 10.1186/s12864-017-4052-4 (PMC5569477; doi:10.1186/s12864-017-4052-4)
Supplement: Supplementary file 1 — Supplementary Figures (S1-S9). (DOCX 2070 kb) [file 12864_2017_4052_MOESM1_ESM.docx]

**Research Article**

**Genome-wide identification of novel intergenic enhancer-like elements: implications in the regulation of transcription in *Plasmodium falciparum***

**Suyog Ubhe^1^, Mukul Rawat^1^, Srikant Verma^2^, Krishanpal Anamika^2^ and**

**Krishanpal Karmodiya^1$^**

^1^Department of Biology, Indian Institute of Science Education and Research, Pashan, Pune 411 021, India.

^2^Persistent Systems Limited Pingala - Aryabhata, Erandwane, Pune 411004, India.

^$^Corresponding author

E-mail: [krish@iiserpune.ac.in](mailto:krish@iiserpune.ac.in)

**Running title: Genome-wide identification of enhancer-like elements**

**Keywords: *Plasmodium falciparum*, Transcription, Enhancers, Histone modifications and Gene Regulation.**

**Supplementary Figure S1.** Graphical representation showing location of 462 Intergenic regions identified over the chromosomes in *Plasmodium falciparum*.

**
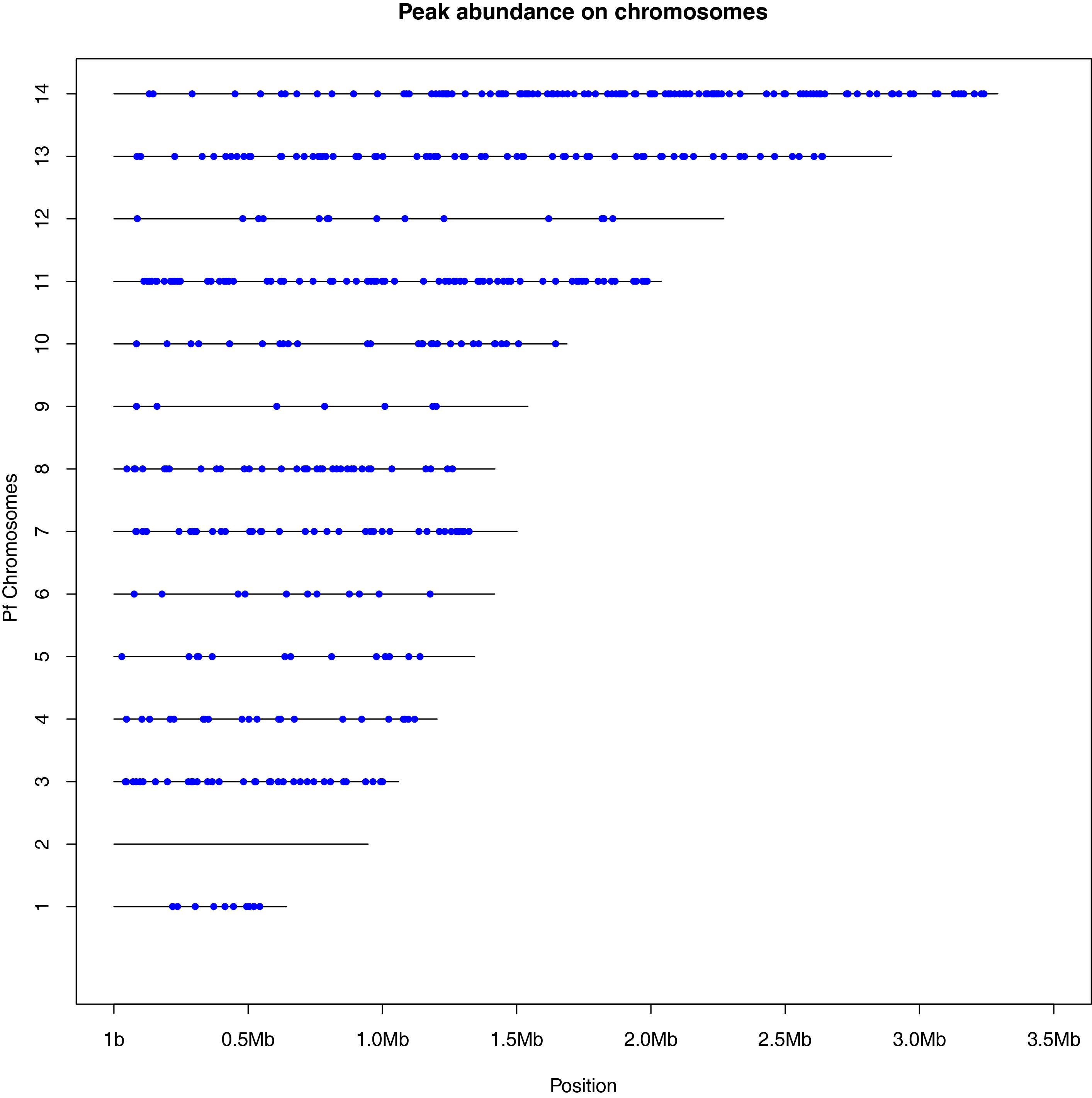
**

**Supplementary Figure S2. Comparison of H3K4me3 modification profiles of the data produced in two different studies.** Normalized mean tag density of H3K4me3 histone modification over 462 intergenic peaks from two different studies, Karmodiya et al., 2015 and Salcedo-Amaya et al., 2009). Enrichment of H3K4me3 on intergenic peaks indicates that our datasets are of comparable quality and the identified IRs are indeed existent.

**
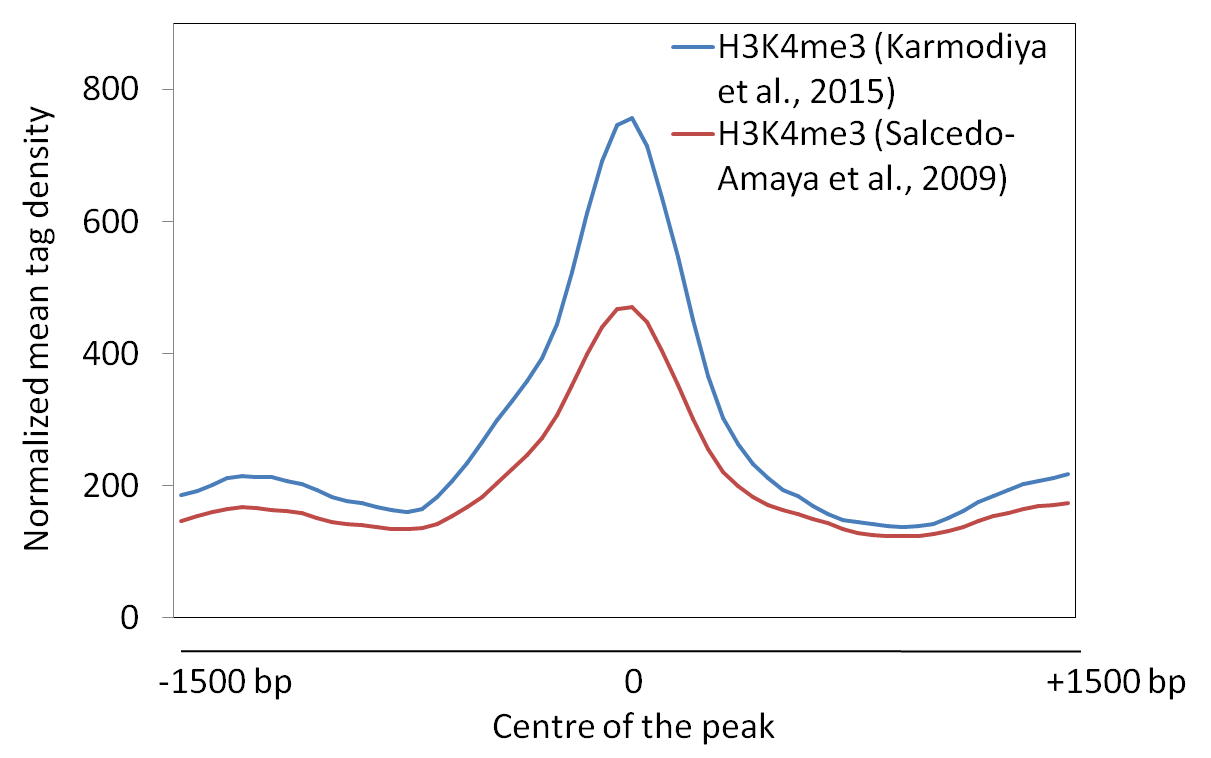
**

**Supplementary Figure S3. Comparison of profiles of H3K4me3 after normalizing with Pan-H3 over identified intergenic** **peaks and promoters.** Profiles of Pan-H3 normalized H3K4me3 over the 462 intergenic peaks and 5265 *Plasmodium* gene promoters. For intergenic peaks centre of the peaks is considered zero. Distribution profile and enrichment of H3K4me3 are comparable over intergenic peaks and promoters suggest that intergenic peaks are not overrepresented regions of the genome because of sequence biases.

**
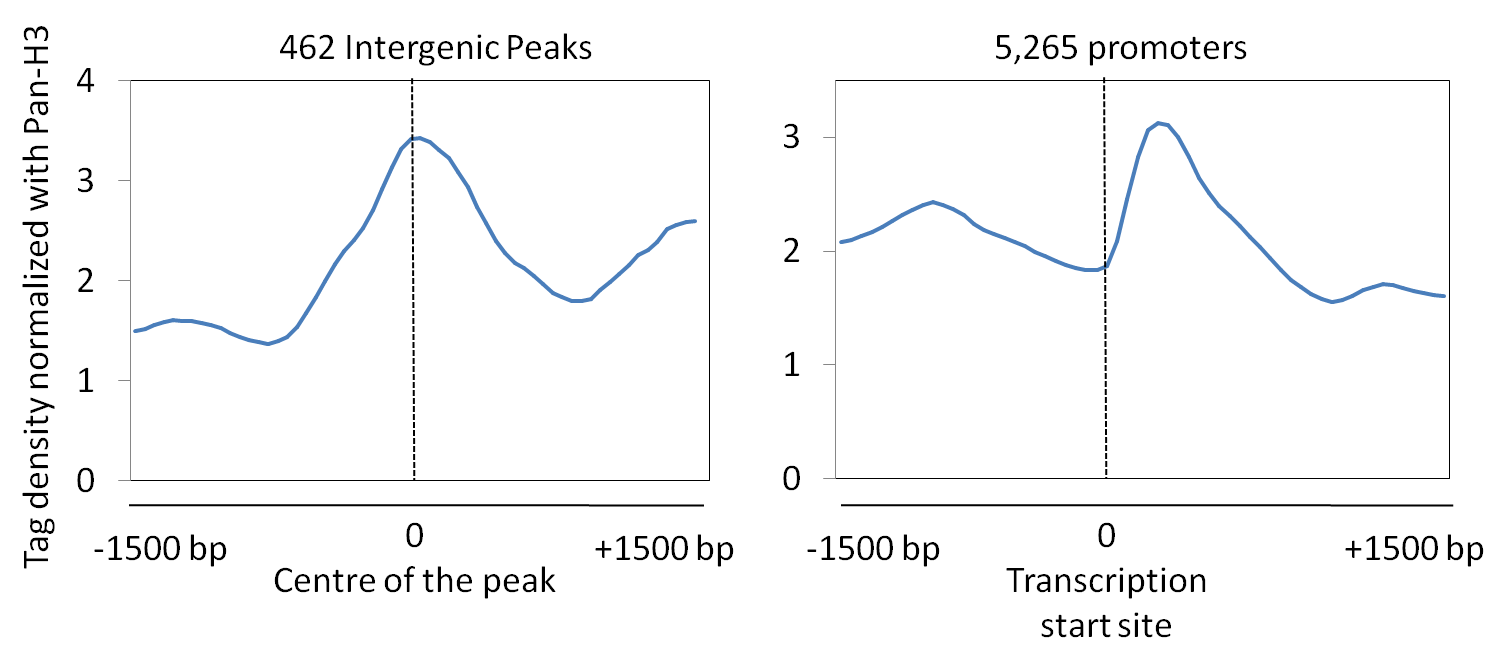
**

**Supplementary Figure S4. Enrichment of histone modifications over intergenic peaks. (A)** Enrichment of H3K36me3, H3K79me3, H4K20me3 and H3K36me2 over the intergenic peaks and 500 strongly and weakly expressed promoters is calculated. H3K36me3, H3K79me3 and H4K20me3 were found comparable at IRs and strong promoters. Surprisingly, H3K36me2 was enriched at promoters. **(B)**Profiles ofH3K36me3, H3K79me3, H4K20me3 and H3K36me2 over the intergenic peaks and 500 strongly and weakly expressed promoters (+/- 0.5kb). Intergenic peaks have bell shaped distribution of all histone modifications. IR: Intergenic regions; SP: Strong Promoters; WP: Weak Promoters.


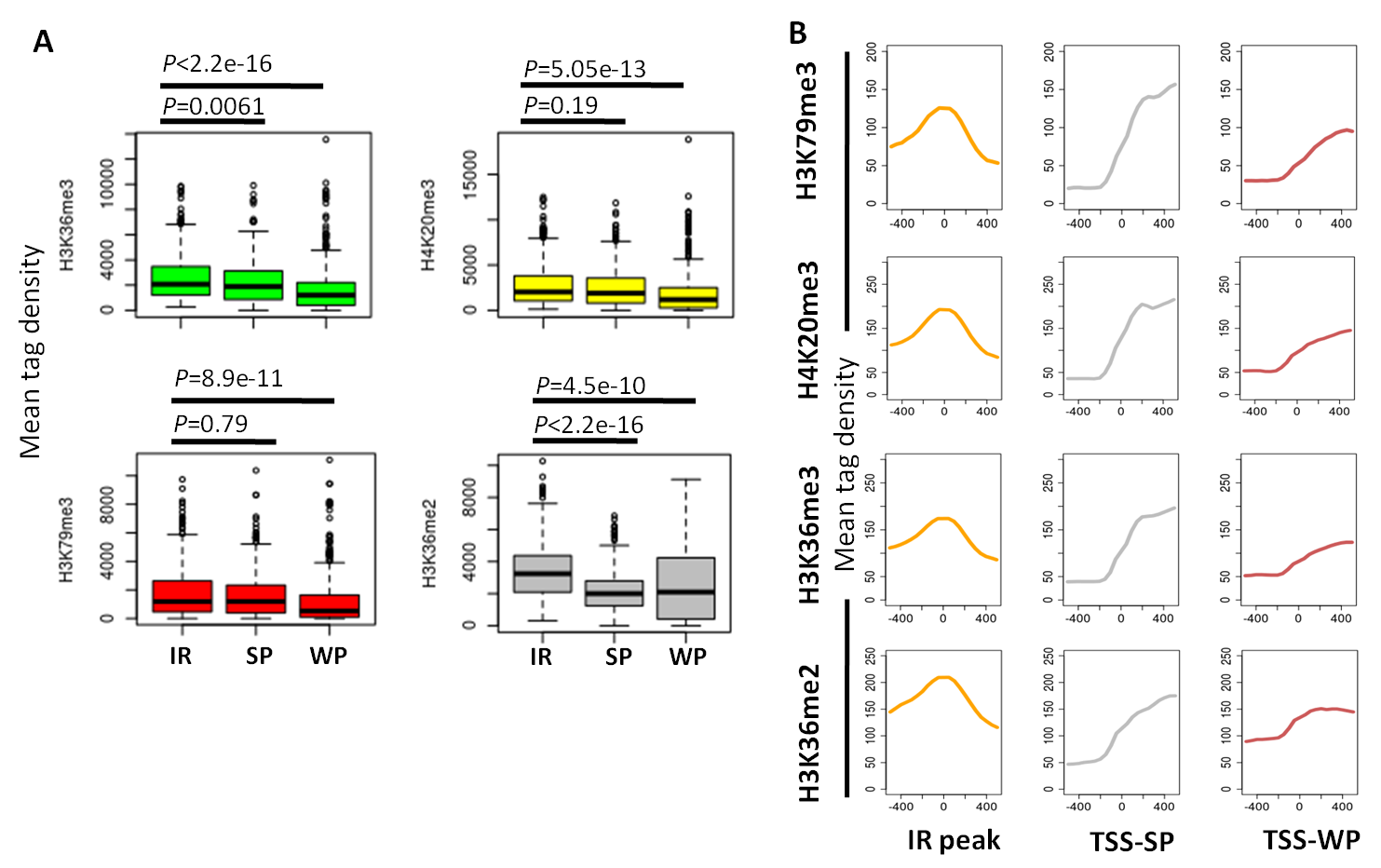


**Supplementary Figure S5. Relative RNA expression from intergenic regions (IRs).** Production of non-coding RNAs form IRs is also verified by (A) qRT-PCR (B) RT-PCR. Relative RNA levels were calculated against a control genomic region. Primer sequences are provided in Supplementary Table S1. Error bars represent the standard deviation calculated from three technical replicates.

**
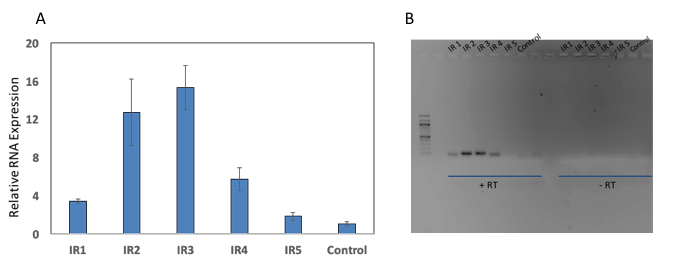
**

**Supplementary Figure S6. Prediction accuracy of coding and non-coding sequences of *Plasmodium falciparum* for evaluating available tools.** Sequences were extracted from PlasmoDB (<http://plasmodb.org>). Pseudogenes were not considered for analysis. There are a total of 5536 non-redundant protein coding genes and 1563 non-coding RNA sequences from multiple *Plasmodium* species. (snRNA, tRNA, rRNA, snoRNA and misc. RNA). CPC was found to accurately predict 99% protein coding and 96% non-coding RNAs from *Plasmodium.*

**Supplementary Figure S7.** Motif enrichment at Intergenic Regulatory Elements (A), Promoters of highly expressed genes (B), and randomly selected intergenic regions (C). Motifs with maximum occurrence, least E-value and unique sequences are presented in Figure 4D.

**Supplementary Figure S8.** TomTom analysis to search for proteins with similar motifs using Campbell, et. al, 2010 database of ApiAp2 motifs. TomTom identified that the DIRE motif – AWGRA has similar recognition sequence as found in PF11_0404_D1, PFL1900w_D1, and PF13_0267 but as indicated the E-values are higher reducing the confidence.

| ApiAP2 | Motif comparison | E-value |
| --- | --- | --- |
| PF11_0404_D1 | 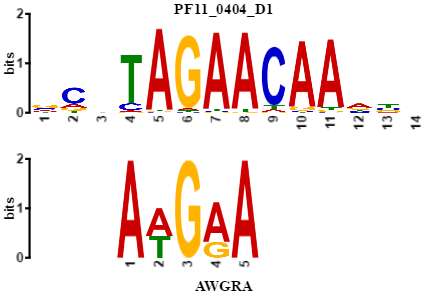 | 6.1e+00 |
| PFL1900w_D1 | 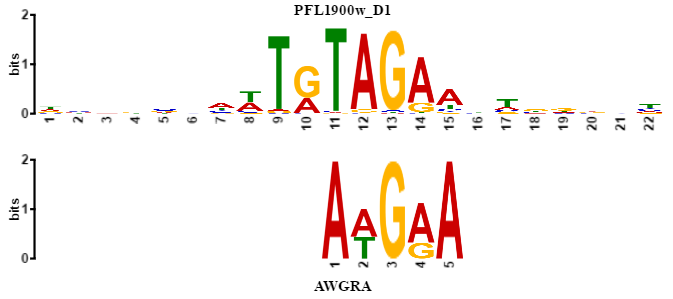 | 7.87e+00 |
| PF13_0267 | 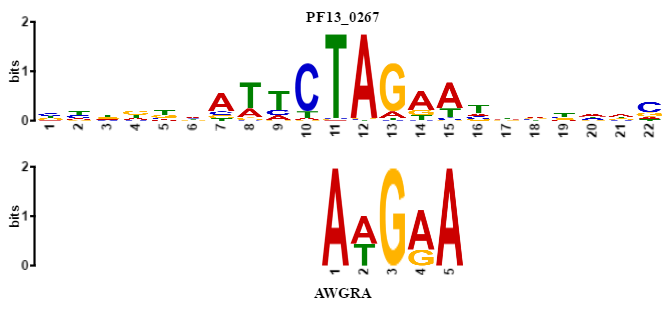 | 8.97e+00 |

**Supplementary Figure S9. Analysis of Hi-C contacts with random genomic sequences.** Equal number (462) of random genomic sequences were taken and analysis was performed for contact information. Total number of contacts in a particular stage was observed less in randomly selected sequences as compared to enhancer-like sequences. The number of contacts drop significantly with higher contacts per gene.

**
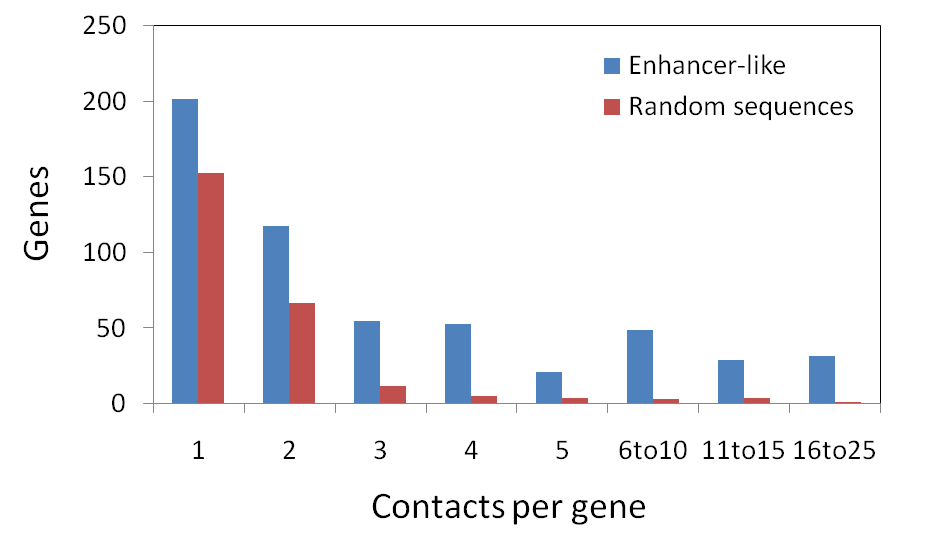
**
